# Machine-to-Machine Transfer Function in Deep Learning-Based Quantitative Ultrasound

**DOI:** 10.1109/TUFFC.2024.3384815

**Published:** 2024-06

**Authors:** Ufuk Soylu, Michael L. Oelze

**Affiliations:** Beckman Institute for Advanced Science and Technology and the Department of Electrical and Computer Engineering, University of Illinois at Urbana–Champaign, Urbana, IL 61801 USA; Beckman Institute for Advanced Science and Technology, the Department of Electrical and Computer Engineering, and the Carle Illinois College of Medicine, University of Illinois at Urbana–Champaign, Urbana, IL 61801 USA

**Keywords:** Biomedical ultrasound imaging, data mismatch, deep learning (DL), tissue characterization, transfer function

## Abstract

A transfer function approach was recently demonstrated to mitigate data mismatches at the acquisition level for a single ultrasound scanner in deep learning (DL)-based quantitative ultrasound (QUS). As a natural progression, we further investigate the transfer function approach and introduce a machine-to-machine (M2M) transfer function, which possesses the ability to mitigate data mismatches at a machine level. This ability opens the door to unprecedented opportunities for reducing DL model development costs, enabling the combination of data from multiple sources or scanners, or facilitating the transfer of DL models between machines. We tested the proposed method utilizing a SonixOne machine and a Verasonics machine with an L9–4 array and an L11–5 array. We conducted two types of acquisitions to obtain calibration data: stable and free-hand, using two different calibration phantoms. Without the proposed method, the mean classification accuracy when applying a model on data acquired from one system to data acquired from another system was 50%, and the mean average area under the receiver operator characteristic (ROC) curve (AUC) was 0.405. With the proposed method, mean accuracy increased to 99%, and the AUC rose to the 0.999. Additional observations include the choice of the calibration phantom led to statistically significant changes in the performance of the proposed method. Moreover, robust implementation inspired by Wiener filtering provided an effective method for transferring the domain from one machine to another machine, and it can succeed using just a single calibration view. Lastly, the proposed method proved effective when a different transducer was used in the test machine.

## Graphical Abstract

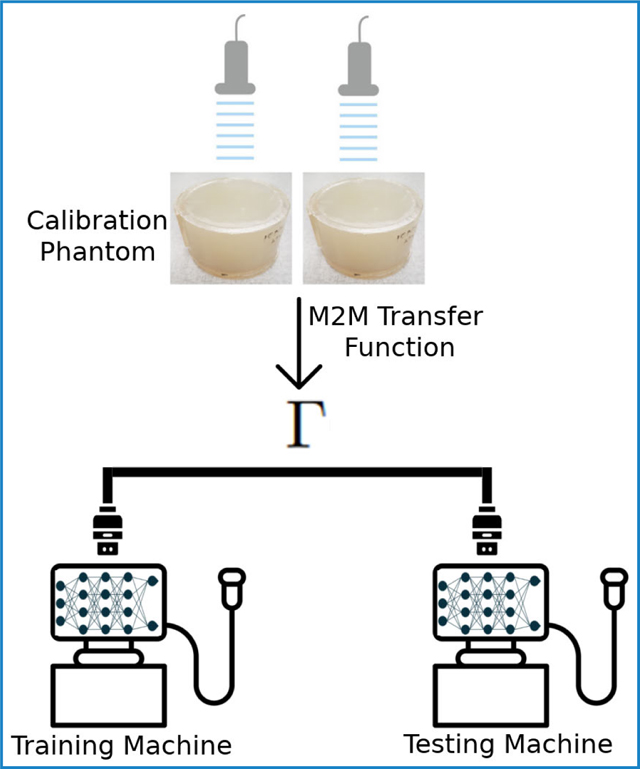

## I. Introduction

In the realm of biomedical ultrasound imaging, deep learning (DL) holds great potential for advancing the field, driven by significant interest from both academia and industry. As DL models become more sophisticated, large datasets become increasingly available, and computational power scales up, the capability of DL to address clinical tasks gets closer to integration into clinical workflows, potentially leading to a transformation in the field of ultrasound imaging. At its essence, DL algorithms learn a sequence of nonlinear transformations, each customizable with parameters, used to derive multiple layers of features from input image data and then make predictions in an automated way, eliminating the need for manual feature extraction. DL is capable of learning high-dimensional functional approximations to perform complex desired behaviors, seemingly defying the curse of dimensionality. Convolutional neural networks (CNNs) emerged as the most preferred and studied approach among DL algorithms in ultrasound biomedical imaging due to their efficiency in analyzing images [1].

The adoption of DL-powered biomedical ultrasound imaging in clinical settings, in an ethical, interpretable, and trustworthy way, is the coveted goal of both industry and the research academy. DL-powered biomedical ultrasound can lead to a significant increase in the quality of medical services in an automated and efficient manner. However, achieving this goal requires major breakthroughs in DL algorithms. Two main technical challenges hinder the implementation of DL-driven algorithms in actual clinical environments [2]. First, for a particular domain, there is often a shortage of labeled data, largely because of the high costs associated with conducting laboratory experiments or obtaining expert annotations from clinical data. Second, the issue of data mismatch arises when the conditions in which a DL model is developed differ from those it will face in clinical setting, which can limit the model’s ability to generalize effectively. In situations where labeled data are scarce or major differences exist between development and deployment environments, any DL-based algorithm might yield poor clinical performance. Consequently, enhancing the data efficacy and robustness of DL algorithms stands as a crucial research direction for establishing DL as a viable tool in ultrasound imaging.

Similar to the general trend in medical imaging, quantitative ultrasound (QUS) has transitioned from classical approaches that rely on manual feature engineering, statistical assumptions, and ad hoc models to DL-based approaches that rely on an abundance of big data and the assumption that training and testing data distributions are identical. Specifically, in several recent examples, CNNs were used to classify tissue states, and it was shown that they outperformed traditional QUS approaches [3], [4], [5], [6], [7], [8]. Following this, in our previous work, a transfer function approach was developed using a calibration phantom to mitigate acquisition-related data mismatches within the same imaging machine for DL-based QUS approaches [9]. The transfer function approach significantly improved mean classification accuracies for pulse frequency, output power, and focal region mismatches within the same imaging machine, increasing them from 52%, 84%, and 85% to 96%, 96%, and 98%, respectively. Therefore, the transfer function approach has emerged as an economical way to generalize a DL model for tissue characterization in cases where scanner settings cannot be fixed, thus improving the robustness of DL-based algorithms.

There is a wide and rich literature anthology related to the data mismatch problem in DL [10], [11]. For example, data augmentation is a crucial tool for minimizing data mismatch. Some approaches build heuristic data augmentations to approximate the distribution shift between testing and training data, aiming to improve robustness [12], [13], [14], [15]. The performance of these approaches depends on how well the approximation mitigates the distribution shift. Other approaches attempt to learn data augmentation by training a generative model between testing and training domains [16], [17], [18], [19]. On the other hand, domain generalization approaches aim to recover feature representations that are independent of domains [20], [21], [22]. Their performance relies on the invariance of the learned features. Additionally, BN-Adapt [23] modifies batch normalization (BN) layers adaptively using test domain data. Moreover, pretraining is another significant concept [24], [25], [26]. Pretraining on a larger dataset could provide robust representations for downstream tasks.

The issue of data mismatch has gained increased attention in recent literature focusing on DL-based QUS [9], [19], [27], [28], [29], [30], [31]. Adaptive BN was utilized in the context of DL-based QUS [27]. Data augmentation with a meta learning algorithm was utilized to generate consistent attenuation coefficient images [31]. In a slightly different line of work, the issue of low sample size for estimating QUS parameters, specifically homodyned-K (HK) parametric images, was addressed using DL-based solutions [32]. Additionally, cycle-consistent generative adversarial networks were applied to address the issue of data mismatches in ultrasound imaging [19]. Furthermore, the Fourier domain adaptation technique was employed, proposing the replacement of lower frequency components within the frequency spectrum [30]. In contrast to these methodologies, the transfer function approach developed in [9] does not require real sample data from the testing domain to be used for training. Instead, it relies on a calibration phantom that can be tailored to the specific characteristics of the real sample at hand. The transfer function separates the calibration process from data acquisition with patients, making the calibration process patient-free from a clinical perspective. Therefore, the transfer function approach provides a practical method to shift the domain of the training dataset to the testing domain, or vice versa, in contrast to these methodologies.

As the transfer function holds the potential for practical implementation within clinical settings, given that it does not necessarily require real samples from the testing domain, it is essential to further validate and identify its strengths and weaknesses under more substantial mismatches. In this study, the application of the transfer function approach was extended to address data mismatches between different imaging machines. By doing so, the transfer function approach would increase its utility in multiple ways. First, being able to transfer between machine domains can lower the cost of DL-based QUS approaches. Specifically, data from different machines can be combined to develop more robust and accurate DL-based models. This has the potential to provide a simple and efficient means of utilizing existing data from different machines and sources, which helps address the high cost associated with labeled data collection. Additionally, DL-based QUS approaches, which are developed for specific machines, can be transferred to other machines at ease. Overall, in our prior work, we demonstrated that the transfer function approach has potential to provide an economical way to provide in-system transferability [9]. In this work, we demonstrate that transfer functions can be defined that can also provide out-system transferability, i.e., a machine-to-machine (M2M) transfer function. Further details of our methodology and experimental results can be found in Sections II and III, respectively. We then provide a section on discussion related to the research findings in Section IV and conclusions in Section V.

## II. Methods

### A. Calibration

In a prior work, a transfer function was developed to mitigate acquisition-related data mismatches within the same imaging machine [9]. In this instance, data mismatch occurs at the machine level, including acquisition and hardware-level mismatches. We follow an identical derivation and notation to develop the M2M transfer function. A simplified decomposition of the frequency spectrum of an ultrasound image involves the system response and the tissue signal [33]

(1) $$I(x,\;f)=S_{\phi }(x,\;f)P(x,\;f)$$

where f represents the frequency, and x represents the axial location. The system response, denoted by $S_{\phi }$, holds the information associated with the ultrasound imaging system and the tissue signal, denoted by P, holds the information associated with the imaging substrate. To mitigate the mismatches between two machines $\phi_{\text{train}}$ and $\phi_{\text{test}}$, we used a calibration phantom P, such that

(2) $$\frac{I_{\text{test}}(x,\;f)}{I_{\text{train}}(x,\;f)}=\frac{S_{\phi_{\text{test}}}(x,\;f)}{S_{\phi_{\text{train}}}(x,\;f)}$$

(3) $$={\text{Γ}}_{\text{train}\to \text{test}}(x,\;f).$$

The M2M transfer function, denoted by ${\text{Γ}}_{\text{train}\to \text{test}}$, is capable of transferring between training and testing machines when the bandwidth is overlapping.

To calibrate a DL network in training time, i.e., train-time calibration (Algorithm 1), ${\text{Γ}}_{\text{train}\to \text{test}}$ can be used

(4) $$I_{\text{train}\to \text{test}}(x,\;f)={\text{Γ}}_{\text{train}\to \text{test}}(x,\;f)I_{\text{train}}(x,\;f)$$

where ${\text{Γ}}_{\text{train}\to \text{test}}$ transformed the training domain into the testing domain. Following that, the model was trained at the test domain directly. This process was referred to as train-time calibration. On the other hand, ${\text{Γ}}_{\text{train}\to \text{test}}^{-1}$(${\text{Γ}}_{\text{test}\to \text{train}}$) can be used for calibrating the DL network in testing time, i.e., test-time calibration (Algorithm 2)

(5) $$I_{\text{test}\to \text{train}}(x,\;f)={\text{Γ}}_{\text{test}\to \text{train}}(x,\;f)I_{\text{test}}(x,\;f).$$

Test-time calibration means that the test set is attempted to be transformed into the training domain through the M2M transfer function so that the originally trained model can be used on the test dataset. Note that the test-time calibration is quicker to implement than train-time calibration because a new model does not need to be trained.

In this work, we investigated two methods for calculating the M2M transfer function. In the first method, stable acquisition was implemented. This involved fixing the transducer using holders and clamps. Following the acquisition of calibration data from one machine, the probe seamlessly transitioned to the other machine without altering its position on the calibration phantom by simply moving the connector from one machine to the other. In the second method, free-hand acquisition was used, involving free-hand motion to record a video of 1000 ultrasound frames from the calibration phantom using both testing and training machines. Additionally, two different types of calibration phantoms, each with uniform scattering properties, were utilized to investigate the effect of calibration phantom selection on calibration performance.

Implementation details of the M2M transfer function are identical to the previous work [9]. The approach taken to incorporate the M2M transfer function drew inspiration from the Wiener filter [34]

(6) $${\text{Γ}}^{\text{Wiener}}=\frac{|\text{Γ}{|}^{-1}}{|\text{Γ}{|}^{-2}+SNR^{-1}}.$$

In the formula above, Γ represents either ${\text{Γ}}_{\text{train}\to \text{test}}$ or ${\text{Γ}}_{\text{test}\to \text{train}}$. SNR was estimated from the power spectra of both $I_{\text{train}}$ and $I_{\text{test}}$ in the calibration data, resulting in $SNR_{\text{train}}$ and $SNR_{\text{test}}$. This was achieved by determining a noise floor level, identified by examining the lowest values of the power spectra, which were at 20 MHz and outside of the analysis bandwidth for the transducers used in the experiments

(7) $${\text{SNR}}_{\text{train}}=\frac{{\left|I_{\text{train}}\right|}^{2}-\min_{f}{\left|I_{\text{train}}\right|}^{2}}{\min_{f}{\left|I_{\text{train}}\right|}^{2}}$$

(8) $${\text{SNR}}_{\text{test}}=\frac{{\left|I_{\text{test}}\right|}^{2}-\min_{f}{\left|I_{\text{test}}\right|}^{2}}{\min_{f}{\left|I_{\text{test}}\right|}^{2}}.$$

Then, the overall SNR was computed for each frequency bin

(9) $$\text{SNR}=\text{min}\left({\text{SNR}}_{\text{train}},\;{\text{SNR}}_{\text{test}}\right).$$

The Wiener implementation offers a robust method, utilizing the complete spectrum of both the train and test domains, resulting in a smoother M2M transfer function. When the SNR is high, the M2M transfer function remains unchanged. However, when the SNR is low, the M2M transfer function behaves as if it was a denoising operation. For simplicity, ${\text{Γ}}_{\text{train}\to \text{test}}$ and ${\text{Γ}}_{\text{test}\to \text{train}}$ represent their Wiener filter implementation version for the rest of this article. Furthermore, the M2M transfer function was computed at different depths to accommodate variations in behavior across the depth range. The calibration techniques are explained in greater detail in Algorithms 1 and 2, where X represents the “patchwise” postbeamformed radio frequency (RF) data, I represents the Fourier spectrum of X, and y represents the phantom identity. Patch extraction will be discussed in Section II–D. During the implementation of experiments, filtering operations due to ${\text{Γ}}_{\text{train}\to \text{test}}$ or ${\text{Γ}}_{\text{test}\to \text{train}}$ were conducted in the time domain. Along with patch data, the axial location was also tracked during patch extraction, resulting in M2M transfer functions having axial location identity as well. Therefore, in the filtering operation, X and its corresponding M2M transfer function based on axial location identity were utilized.

**Algorithm 1:** Train-Time Calibration

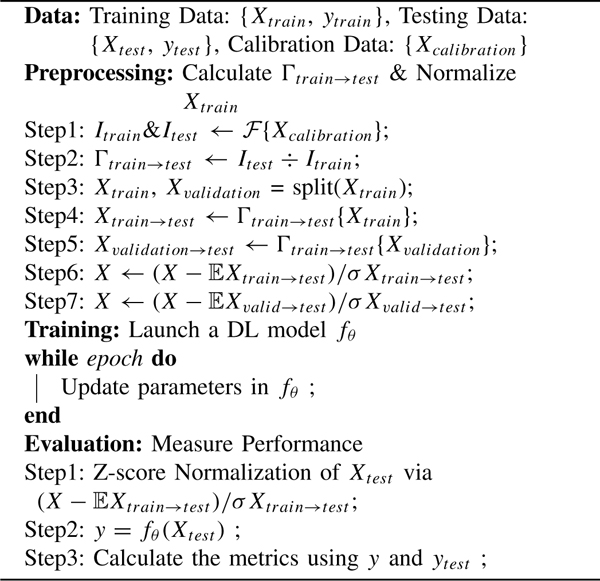

Z-score normalization/standardization was carried out at the patch level as a default data preprocessing step for training and evaluating the DL models. This process includes subtracting the mean patch, then dividing by the standard deviation patch. In Algorithm 1, during the evaluation stage, statistics from $X_{\text{train}\to \text{test}}$ were obtained and used for z-score normalization. On the other hand, in Algorithm 2, statistics from $X_{\text{train}}$ were used at z-score normalization. Therefore, Algorithms 1 and 2 do not rely on any information from $X_{\text{test}}$, making the M2M transfer function practical and economically feasible for clinical implementation.

In addition, in Algorithms 1 and 2, during both training and evaluation, BN layers were modified to operate with batch statistics, i.e., in PyTorch, and running statistics of BN layers were set to None. Under this condition, BN layers have learnable affine parameters, but they utilize batch statistics instead of updating running statistics. Hence, in these algorithms, affine parameters in BN layers were learned utilizing batch statistics.

### B. Phantoms

**Fig. 1.**
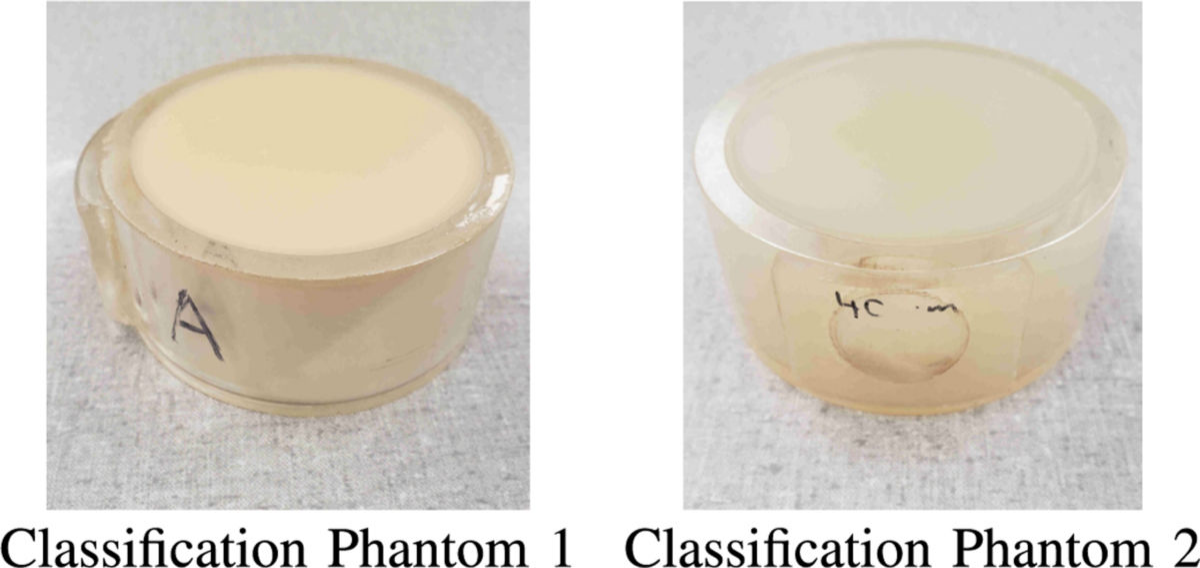
Visuals of the classification phantoms.

**Fig. 2.**
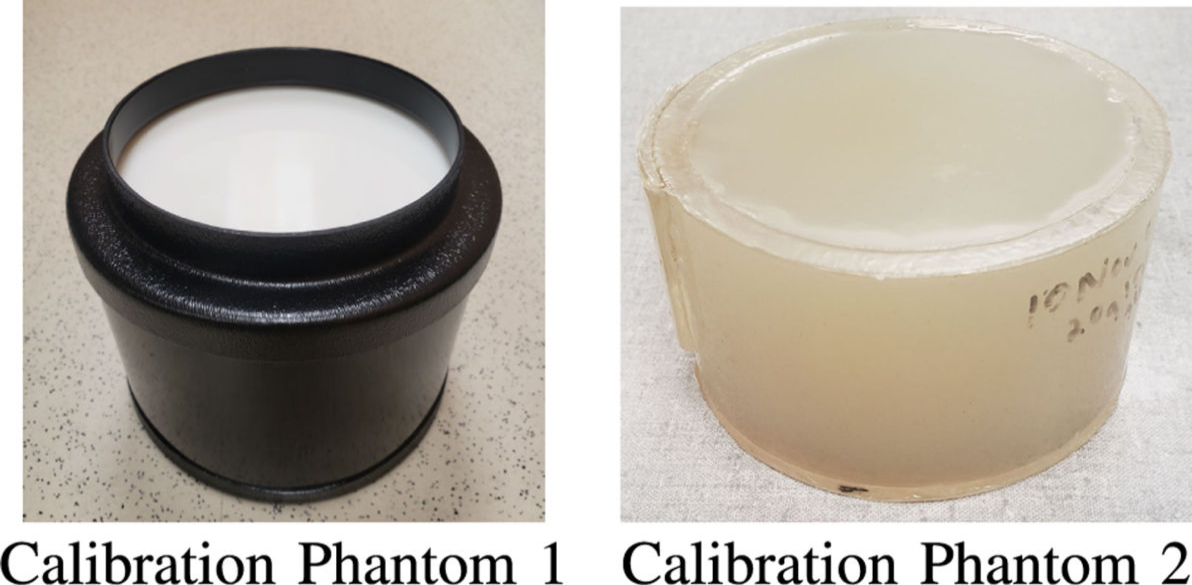
Visuals of the calibration phantoms.

The experiments utilized two distinct tissue-mimicking phantoms as classification phantoms, as shown in Fig. 1. Additionally, two distinct calibration phantoms were used to obtain the M2M transfer function, as shown in Fig. 2 and summarized in Table I. In Table I, speed of sound (SOS) is given in terms of m × s^−1^, attenuation (Atten) is given in terms of dB × cm^−1^ × MHz^−1^, bead diameter (BD) is given in terms of *μ*m, and bead concentration (BC) is given in terms of g × L^−1^. The beads were glass spheres with selected mean sizes and distributions to act as scatterers in the phantoms.

**Algorithm 2:** Test-Time Calibration

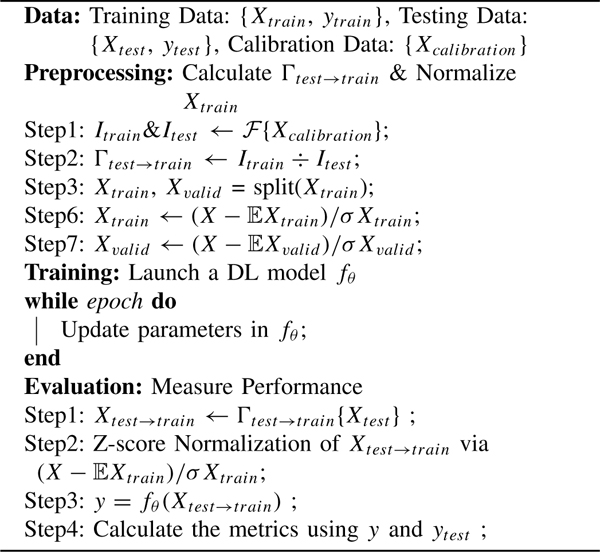

Classification Phantom 1 mimics the characteristics of the human liver [35] and the construction details were given in [36]. The attenuation coefficient slope for Classification Phantom 1 was measured as 0.4 dB × cm^−1^ × MHz ^−1^. It exhibited macroscopic uniformity. The speckle pattern in Classification Phantom 1 stemmed from the random distribution of microscopic glass bead scatterers, ranging in diameter from 82.5 ± 7.5 *μ*m. Its SOS was 1540 m × s^−1^.

Classification Phantom 2 was characterized as a low-attenuation phantom [37], and the construction details were given in [38]. The same weakly-scattering agar, serving as the background material, was utilized in Classification Phantom 2 but included glass-bead scatterers of varying sizes, ranging from 41 ± 2 *μ*m in diameter. Its SOS was 1539 m × s^−1^. The attenuation coefficient slope was measured as 0.1 dB × cm^−1^ × MHz^−1^.

The Calibration Phantom 1 was a commercial QUS reference phantom (part no. 14090502, serial no. 221447541) from CIRS, Inc., Norfolk, VA, USA. It had an attenuation coefficient slope of 0.74 dB × cm^−1^ × MHz ^−1^. Its SOS was 1545 m × s^−1^.

The Calibration Phantom 2 was characterized as a low-attenuation phantom [37]. We used the approach by Anderson et al. [37] who utilized the arrival time difference [39], the insertion loss techniques [35], and the narrowband through-transmission technique [38] to characterize SOS and attenuation. It was constructed with a 2% agar background having weakly scattering properties. This phantom included glass beads with diameters measuring 160 ± 60 *μ*m. The distribution of glass beads, occurring spatially randomly within the phantom’s volume, was at a concentration of 20 g/L. The attenuation coefficient slope for Classification Phantom 2 measured as 0.6 dB × cm^−1^ × MHz^−1^. Its SOS was 1535 m × s^−1^.

### C. Ultrasound Machines

The phantoms were scanned using both a SonixOne system and a Verasonics Vantage 128. An Ultrasonix L9–4 transducer and a Verasonics L11–5 transducer were utilized throughout the experiments. The datasets are summarized in Table II. The SonixOne system captured postbeamformed RF data with a sampling rate of 40 MHz. Under the hood, the raw RF channel data had a 16 bit depth, and the beamforming operation was performed with a 64 bit depth in the SonixOne machine. In contrast, the Verasonics system acquired raw channel data and did not return postbeamformed data. The Verasonics data were sampled at a rate of 50 MHz with a 16 bit depth. Subsequently, delay and sum beamforming were implemented on the Verasonics data using a 64 bit depth, similar to the SonixOne system. Following this, a multirate finite-impulse response (FIR) filter was designed with an interpolation factor of 4 and a decimation factor of 5 to convert the sampling rate to 40 MHz. Beamforming and sampling rate conversion were implemented using MATLAB (version: R2023a) functions. Specifically, the “designMultirateFIR” function was used, which computes the filter coefficients based on the interpolation and decimation factors, while the “dsp.FIRRateConverter” function was used to implement a combined antialiasing FIR filter using these filter coefficients, the decimation factor, and the interpolation factor. After these preprocessing steps, postbeamformed RF data at a matching sampling frequency of 40 MHz were obtained from both machines for DL operations. Matching the sampling rate between systems was critical to being able to implement the M2M transfer function.

As training data, the SonixOne data, acquired with L9–4 transducer, were utilized during the experiments, positioning the SonixOne as the “training machine” where the model development occurred. On the other hand, the Verasonics data, acquired with both L9–4 and L11–5 transducers, were utilized as testing data during the experiments, positioning it as the “testing machine” where the machine data are assumed to be unavailable during model development. Testing machine data were only used to measure calibration success during inference time. For training data and testing data, free-hand data acquisition was utilized with Classification Phantom 1 and Classification Phantom 2, i.e., the transducer was moved across the phantom surface by hand. During this acquisition, by recording a video of 1000 frames, we captured a large amount of ultrasound data for each phantom.

For calibration data, both the SonixOne and Verasonics machines were utilized in two scanning procedures using Calibration Phantom 1 and Calibration Phantom 2. In the first procedure, similar to the training and testing data, free-hand acquisition was utilized, which provided 1000 independent frames from each calibration phantom. The second procedure, termed stable acquisition, involved securing the transducer using a bar clamp holder. Subsequently, ten identical frames were captured using both the SonixOne and Verasonics machines from precisely the same position on the calibration phantoms. These procedures facilitated the acquisition of calibration data necessary for computing the M2M transfer function.

As imaging settings, line-by-line acquisition with 2-cm axial focal point at transmission with dynamic focusing during reception was used for both machines. In the SonixOne, the center pulse frequency was set at 9 MHz and its output power level was set at 0 dB. In the Verasonics, the center pulse frequency was set at 5 MHz and its output power level was set at 45.2 V. These settings were configured to evaluate the proposed method under combined hardware and acquisition-related mismatches.

### D. Data Preparation

An ultrasound image frame size from both the testing and training machines was 2080 pixels × 256 pixels after all processing. The axial depth was 4 cm. After obtaining frames, square data patches from the frames were extracted to be employed in the DL network. These patches measured 200 samples × 26 samples, corresponding to physical dimensions of 4 × 4 mm. The motivation for patch extraction is rooted in traditional QUS approaches. In traditional tissue characterization, a data patch is extracted from the ultrasound image to examine the ultrasound signals within a region of interest. The input of the DL network consisted of raw backscattered RF data. More specifically, “patchwise” data of z-score-normalized RF signals were used at its input.

**Fig. 3.**
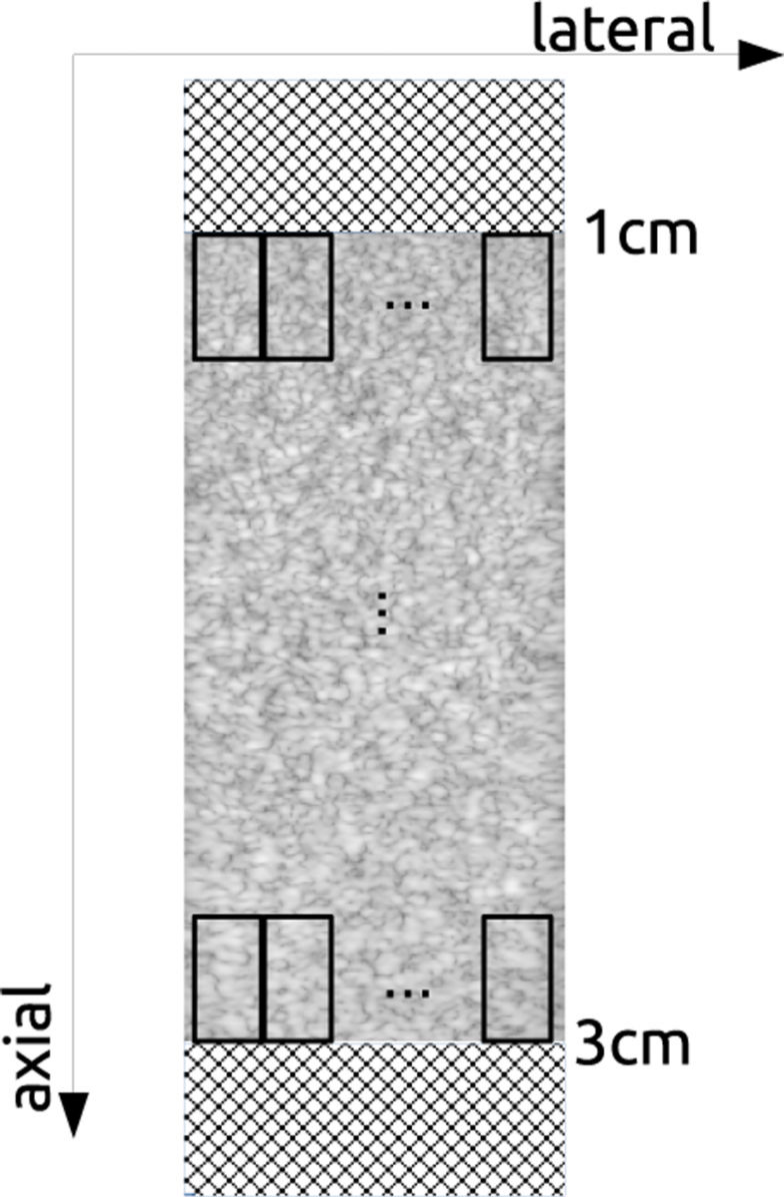
Process of patch extraction in data preparation.

We were able to extract 81 image patches (nine lateral positions and nine axial positions) from a single frame, as illustrated in Fig. 3. During the extraction process, the initial 540 axial pixels were omitted. The next sequence of patches in the lateral direction was generated by moving the beginning of the succeeding patch by 26 pixels. In the axial direction, the next sequence of patches was generated by moving the beginning of the succeeding patch by 100 pixels. This method resulted in nine patches axially by nine patches laterally, allowing us to extract a total of 81 image patches from each ultrasound image. During patch extraction, in addition to patch data, the axial location and the class identity were also tracked for the purposes of training, calibration, and evaluation. Each data point was composed of three entities: ultrasound patch, axial location ranging from 0 to 8 (where 0 represents the first axial line and 8 represents the last axial line), along with the class identity.

From the training machine, 2000 ultrasound frames were acquired, with 1000 frames from each classification phantom, to be used in training, resulting in 162000 patches. From the testing machine, 1000 ultrasound frames were acquired, with 500 frames from each classification phantom, to be used in testing, leading to 81000 patches. Regarding calibration data, through stable acquisition, ten frames from a fixed point were acquired for each machine, and through free-hand acquisition, 1000 frames were acquired for each machine.

In the experimental setup, a balanced configuration was investigated in terms of both class identify and depth location. During partitioning the data into training and validation sets, data points were uniformly and randomly grouped into two sets, with the training set representing four-fifth of the total patches. The setup ensured equal representation of patches from different depths and different classes in training, validation, and calibration.

### E. Training

The DL algorithms were trained utilizing a workstation equipped with four NVIDIA RTX A4000. Each experiment was conducted using all four RTX A4000s in parallel. The PyTorch library [40] was utilized for all experiments.

In all experiments, we utilized the Adam algorithm [41] as the optimizer. Hyperparameters, including epoch numbers and learning rates, were determined aiming for “asymptotic test accuracy.” The batch size was selected as 2048 to maximize memory utilization. During training, a standard method for data augmentation involved applying a horizontal flip with a 50% probability by default. As training loss, cross-entropy loss was utilized. In the training phase, the data patches were split into training and validation sets using a uniform random approach, with a ratio of 4:1.

Each experiment, i.e., the training, was repeated ten times. Next, the average of the classification accuracies, the average area under the receiver operator characteristic (ROC) curve (AUC) and their respective standard deviations were computed using the test sets. The results were obtained patchwise. The variance in the results was caused by the random initialization of network parameters at each repetition. In the code, random seed was included, ensuring that the results were reproducible.

### F. Network Structure

We employed two established CNN architectures in this study: ResNet-50 [12] and DenseNet-201 [42]. We made minor adjustments to the CNN architectures to customize their input–output relationship to suit our specific problem. The first convolutional layers, which originally took three input feature channels, were replaced with a single input-channel convolution layer. Additionally, the last layer, a fully connected layer, was also modified to output a single probability corresponding to two classes. For network parameter initialization, pretrained weights were used, except for the first convolutional layer and the last fully connected layer, which were initialized using the default method in PyTorch. During training, all the parameters were unfrozen and fine-tuned through backpropagation.

### G. Fine-Tuning

Pretraining is a significant concept in DL. Pretraining on a larger dataset could provide robust representations for downstream tasks in scenarios with low data availability. In this case, pretraining on the training machine data was followed by fine-tuning with a smaller dataset from the test machine, offering an efficient approach to overcoming machine-level data mismatches. Therefore, as a baseline method, the proposed approach was compared with the fine-tuning approach. In the fine-tuning approach, after the pretraining stage, 100 ultrasound frames from the testing machine were utilized to fine-tune the pretrained model. It is important to note that this approach requires diverse frames from the testing conditions, and the data must be from the classification phantoms. Moreover, during the fine-tuning stage, for ResNet, the last three Conv2D layers and the linear layer were unfrozen. For DenseNet, the last four Conv2D layers and the linear layers were unfrozen.

### H. AUC Analysis

Another interesting baseline involves conducting AUC analysis on the pretrained model. In this approach, instead of fine-tuning the pretrained model, AUC analysis was performed with a small dataset from the testing machine to identify the optimal threshold for the last logit (after the sigmoid) outputted by the DL network. It is assumed that under the mismatch, the pretrained model remains robust in terms of separability, indicated by a high AUC score in testing conditions. The main degradation is expected to arise from shifting the threshold to determine the class identity in the last logit. Therefore, the proposed method was compared with the AUC analysis. It is important to note once again that this approach has similar limitations to fine-tuning, requiring diverse frames from the testing conditions, and the data must be from the classification phantoms. In the AUC analysis approach, 100 ultrasound frames were utilized.

### I. BN Freezing

It has been demonstrated that BN layers play a critical role in preserving domain information [43]. Subsequently, in one line of domain adaptation techniques, BN layers were modified and adapted to learn domain invariant features. In ultrasound imaging, Tehrani et al. [29] demonstrated that freezing BN layers during training improves domain adaptation. Therefore, as a third baseline method, the proposed calibration approach was compared to BN freezing. However, it is important to note that even though there is no direct usage of any information related to the test data during training, at evaluation time, test statistics were needed to properly apply z-score normalization. Hence, the only limitation of this approach, in comparison to the other two baseline methods, is the assumption that z-score normalization statistics at test time are given.

### J. No Calibration

The proposed method was also compared to the scenario where no calibration or adaptation method was implemented. In the results, three experiments were conducted under the no-calibration case: 1) No calibration; 2) No calibration but z-score calibrated statistics; and 3) No calibration but z-score test statistics. Different statistics represent different z-score normalization statistics in the input during the evaluation stage. “No calibration” utilizes $EX_{\text{train}}$ and $\sigma X_{\text{train}}$. This experiment represents the worst case, where no mismatch mitigation is applied. “No calibration but z-score calibrated stats” utilizes $EX_{\text{train}\to \text{test}}$ and $\sigma X_{\text{train}\to \text{test}}$. This experiment represents the use of the proposed calibration method in its simplest form by altering z-score normalization statistics in the input only. “No calibration but z-score test stats” utilizes $EX_{\text{test}}$ and $\sigma X_{\text{test}}$. This experiment represents the perfect adaptation of the input normalization, relying on true information of test data statistics, which refers to an idealized experiment, serving as a reference for comparison.

### K. Statistical Test

As a statistical test, the Wilcoxon signed-rank test was utilized using the scipy.stats.wilcoxon function from the SciPy library. In the results, p-values for each pair are presented in tabular format, where the right upper side (above the diagonal) was reserved for ResNet, and the lower left side (below the diagonal) was reserved for DenseNet. Moreover, ∗ represents not applicable scenarios and < 0.01 represents statistically different pairs at threshold *p* = 0.01.

## III. Results

### A. Transfer Function Versus Baseline Methods

We compare train-time calibration (Algorithm 1), test-time calibration (Algorithm 2), no calibration experiments, and the baseline methods in Table III. Statistical tests are given in Tables IV and V. In these experiments, stable acquisition with Calibration Phantom 1 was utilized to obtain the M2M transfer function. We set the learning rate to 1e−5 and the number of epochs to 50 for both train-time and test-time calibration. For the no calibration experiments, we set the learning rate to 5e−6, and we ran the training for 25 epochs to facilitate early stopping. For the fine-tuning, after the pretraining stage which was no calibration case, we set the learning rate to 1e−5 and the number of epochs to 50. For the AUC analysis, there was no extra training over no calibration case. Moreover, for the BN freezing, we set the learning rate to 1e−5 and the number of epochs to 50. The results reveal a significant gain achieved by the proposed method. Furthermore, the proposed method offers additional advantages in comparison to the baseline methods, such as not requiring any data from classification samples and needing only a single stable view from a calibration phantom to calculate the transfer function.

### B. Different Calibration Phantoms

We investigated the effects of using different calibration phantoms, Calibration Phantom 1 and Calibration Phantom 2, on the success of calibration, as shown in Table VI. The results of statistical tests are provided in Tables VII and VIII. For train-time calibration and test-time calibration, we set the learning rate to 1e−5, and ran the training for 50 epochs. Stable acquisition was utilized in these results because we only used a single transducer, the L9–4, for both machines. Utilizing different calibration phantoms led to statistically different behavior for both train-time and test-time calibrations.

### C. Stable Versus Hand-Free Calibration

We investigated the effects of using different acquisitions for the calibration data, stable and free-hand, on the success of calibration, as shown in Table IX. Statistical tests are provided in Tables X and XI. For train-time calibration and test-time calibration, we set the learning rate to 1e−5, and ran the training for 50 epochs. Calibration Phantom 1 was utilized in these results. Utilizing free-hand calibration did not result in performance improvement over stable calibration.

### D. Different Transducers

We investigated the effects of using different transducers for the test machine on the success of calibration, as shown in Table XII. Statistical tests are provided in Tables XIII and XIV. For the training machine, the L9–4 was utilized while the L11–5 was utilized for the testing machine. For train-time calibration, this effect did not yield any statistically significant differences, while for test-time calibration, it led to statistically different results. When using two separate transducers, only free-hand calibration could be used.

## IV. Discussion

The M2M transfer function has the potential to be implemented in practice as it does not rely on the acquisition of test domain data from classification samples to calibrate the classifier. The approach can provide a practical means to transfer DL models between imaging machines and to transfer data from different sources to the desired domain, thereby significantly reducing model development costs. In this article, an M2M transfer function was investigated using different calibration phantoms and different acquisition strategies for acquiring calibration data. Along with the M2M transfer function, BN layers were modified to utilize batch statistics at train and evaluation. We observed that the M2M transfer function was effective in calibrating a DL model between imaging machines, increasing mean classification accuracy from 50.01% to 98.73% and mean AUC from 0.405 to 0.999 for ResNet architecture; and increasing mean accuracy from 47.19% to 98.96% and mean AUC from 0.488 to 0.999 for DenseNet architecture.

In Table III, we mainly observe that the M2M transfer function provided perfect calibration under machine-level data mismatches. Compared to the baseline methods, the proposed method achieved a significant performance improvement. Additionally, there were multiple interesting observations that can be derived from Table III. First, in the case of no calibration, the use of training statistics resulted in very poor performance, as expected. However, the utilization of calibrated statistics and test statistics led to a significant improvement in accuracy and AUC. The accuracy improved from 50% to the range of 70%–75%, and the AUC increased from 0.5 to above 0.9. It is worth noting that a significant improvement was achieved by using calibrated statistics even without calibrating input data. This improvement was observed solely by implementing calibration for the statistics used in the normalization step, demonstrating the potential of the M2M transfer function.

Another important observation from Table III is that the test-time calibration performed significantly better than train-time calibration in terms of accuracy and AUC. This observation differs from previous work on the setting transfer function and was achieved after BN layer modification (choice of the BN). Specifically, BN layers were updated to operate with batch statistics both during training and evaluation. However, further study is needed to understand the performance differences between train-time and test-time. In terms of network architecture, it was found that the proposed method was effective for both ResNet and DenseNet and test-time calibration provided perfect calibration for both architectures. In comparison to the baselines, the proposed method provided significant performance improvement in terms of both accuracy and AUC. It is noteworthy that the performance of Experiment 4, fine-tuning, and BN freezing were statistically insignificant in terms of AUC for the ResNet architecture, verifying the usefulness of the proposed method.

Given the best-performing approach, the calibration approach holds other advantages over the baseline methods. The proposed method does not require data acquisition from classification samples from the testing machines. Instead, it only requires a single calibration view from a calibration phantom. This aspect of the proposed method, especially, positions it as a practical and clinically viable method.

In Table VI, Calibration Phantom 1 and Calibration Phantom 2 were used for both train-time and test-time calibrations. We observed that Calibration Phantom 1 resulted in better calibration for both train-time calibration and test-time calibration in terms of accuracy and AUC. These results suggest that the selection of a calibration phantom was relevant to performance. Even though the proposed method did not rely on the acquisition of real samples from the test domain, one could hypothesize that the calibration phantom selection should align with the classification samples. As known from standard QUS [33], the properties of the calibration phantom, specifically SOS and attenuation, should resemble those of the test and training domain samples to enhance the calibration process.

Another interesting aspect of calibration phantom selection is the SNR values obtained for (6). Through qualitative investigation of the overall SNR values obtained from ultrasound signals from the calibration phantoms, we found that Calibration Phantom 2 had a higher SNR for lower frequencies, while Calibration Phantom 1 had a higher SNR for higher frequencies. Therefore, one might conclude that the calibration of higher frequencies was more important for calibration performance than the calibration of lower frequencies. However, further studies in DL-based approaches should be conducted to develop a systematic approach for selecting a calibration phantom based on properties of the real sample in the training domain, which is known.

In Table IX, stable acquisition and free-hand acquisition were investigated in terms of calibration performance. In stable acquisition, the M2M transfer function was calculated using a single fixed view from the calibration phantom. In free-hand acquisition, a video of ultrasound frames was recorded, and the M2M transfer function was calculated by averaging it over the frames. The results indicate that free-hand calibration does not have any advantage over stable calibration. This may sound counter intuitive at first as free-hand acquisition uses more frames to calculate an M2M transfer function; however, this observation actually verifies the robustness of the Wiener-inspired implementation against noise. Apparently, the Wiener-inspired implementation provides a robust method to calibrate data mismatches using just a single, fixed ultrasound frame. That being said, as a future direction, utilizing multiple calibration views to enhance calibration performance still remains an attractive avenue.

In Table XII, the calibration method was tested against a mismatch of the transducer at the testing machine. It was observed that, during train-time calibration, the use of a different transducer at the testing machine did not yield any statistically relevant change assuming the bandwidths were similar. However, for test-time calibration, a 1%–1.5% accuracy loss and a 0.002–0.003 AUC score loss occurred. This verifies the robustness of the proposed approach under transducer mismatch; however, further study will be needed to enhance our understanding.

The results of this work highlight several potential future directions. First, the findings suggest that the selection of a calibration phantom can significantly impact performance. Specifically, the SOS and attenuation of the calibration phantom are expected to play a critical role. In this article, SOS characteristics varied between 1535 and 1545 m × s^−1^ and attenuation characteristics varied between 0.1 and 0.7 dB × cm^−1^ × MHz^−1^. As a future direction, expanding these ranges and observing their effects on calibration performance would lead to a systematic approach for choosing a calibration phantom. Therefore, developing a systematic procedure for choosing a calibration phantom remains an important problem. Second, enhancing calibration performance by leveraging multiple calibration views remains unsolved. Although in this work, free-hand calibration could not provide additional performance improvement, utilizing multiple views from a calibration phantom could be effective in certain scenarios. Third, the impact of using different transducers, and thus, different usable frequency ranges on calibration, and devising solutions to address potential challenges arising from variations in transducer bandwidth, requires additional study. Even though the proposed approach works effectively between an L9–4 transducer and an L11–5 transducer, it is expected that the technique will break down as the bandwidth overlap between systems and their respective transducers is reduced. One could try to match the ranges and/or implement the transfer function over the intersecting frequencies. Similarly, the effects of different acquisition techniques, such as plane wave imaging versus line-by-line imaging or even changes in sampling rate, on the calibration may also affect the ability to transfer classification models from one machine to another. Another intriguing aspect is the digitization bit. In this work, digitization bits were matched between domains. However, in cases of strong quantization artifacts or highly scattering materials, the calibration performance may be affected. Finally, from a security perspective, in scenarios where DL model transferability is not desired, it may be possible to develop defense mechanisms, such as introducing sampling rate mismatches along with mismatched digitization bits. Moreover, if data acquired from multiple machines can be combined through an M2M transfer function, the increase in data availability, i.e., incorporating the data from multiple machines, could lead to improved DL models. The code for the implementation of training, testing, and calibration can be accessed at the following repository: https://github.com/usoylu2/m2m. The dataset is available for use via the following link: https://uofi.box.com/s/d9ecw002ree6gj9tlplz7t0i2f1ojbk7.

## V. Conclusion

We introduced an M2M transfer function for mitigating the effects of data mismatches between data acquired from different ultrasound scanners. The results indicate that the M2M transfer function can be effective in calibrating mismatches between different imaging machines. Therefore, the incorporation of M2M transfer function can offer an economical approach to transferring datasets and DL models between machines, reducing the cost of model development and paving the way for an enhanced understanding of model security.

## Figures and Tables

**TABLE I:** Phantoms

*Phantoms*	SOS	Atten	BD	BC
Class. Ph. 1	1540	0.4	82.5±7.5	3.8
Class. Ph. 2	1539	0.1	41±2	2.23
Calib. Ph. 1	1545	0.7	Unknown	Unknown
Calib. Ph. 2	1535	0.6	160±60	2.23

**TABLE II:** Imaging Conditions

*No*	Machine	Trans.	Type	Freq.	Sampling	Ampl.
1	SonixOne	L9–4	training	9MHz	50MHz	0dB
2	Verasonics	L9–4	testing	5MHz	40MHz	45.2V
3	Verasonics	L11–5	testing	5MHz	40MHz	45.2V

**TABLE III:** Transfer Function Versus Baseline Methods

No	Experiment	ResNet-Acc.	DenseNet-Acc.	ResNet-AUC	DenseNet-AUC
1	Train-Time Calibration	93.69±0.70	94.36±0.97	0.983±0.003	0.984±0.004
2	Test-Time Calibration	98.73±0.30	98.96±0.32	0.999±4e-4	0.999±4e-4
3	No Calib	50.01±0.01	47.19±5.94	0.405±0.073	0.488±0.157
4	No Calib. but Calib. Stats	70.59±4.73	68.40±2.86	0.936±0.021	0.919±0.017
5	No Calib. but Test Stats	75.11 ±4.67	72.65±3.23	0.949±0.011	0.936±0.139
6	Fine-Tuning	86.32±1.12	86.82±1.06	0.946±0.009	0.945±0.008
7	AUC Analysis	87.94±0.90	85.96±2.24	0.950±0.010	0.937±0.014
8	BN Freezing	87.97±1.41	89.46±0.72	0.95Ü0.011	0.963±0.005

**TABLE IV:** *p*-Values for Table III Based on Classification Accuracy

*No*	1	2	3	4	5	6	7	8
1	*	<.01	<.01	<.01	<.01	<.01	<.01	<.01
2	<.01	*	<.01	<.01	<.01	<.01	<.01	<.01
3	<.01	<.01	*	<.01	<.01	<.01	<.01	<.01
4	<.01	<.01	<.01	*	<.01	<.01	<.01	<.01
5	<.01	<.01	<.01	<.01	*	<.01	<.01	<.01
6	<.01	<.01	<.01	<.01	<.01	*	<.01	.06
7	<.01	<.01	<.01	<.01	<.01	.43	*	.77
8	<.01	<.01	<.01	<.01	<.01	<.01	<.01	*

**TABLE V:** *p*-Values for Table III Based on AUC Scores

*No*	1	2	3	4	5	6	7	8
1	*	<.01	<.01	<.01	<.01	<.01	<.01	<.01
2	<.01	*	<.01	<.01	<.01	<.01	<.01	<.01
3	<.01	<.01	*	<.01	<.01	<.01	<.01	<.01
4	<.01	<.01	<.01	*	<.01	.38	<.01	.04
5	<.01	<.01	<.01	<.01	*	.56	.13	.43
6	<.01	<.01	<.01	<.01	.01	*	.32	.37
7	<.01	<.01	<.01	<.01	.08	.02	*	.63
8	<.01	<.01	<.01	<.01	<.01	<.01	<.01	*

**TABLE VI:** Different Calibration Phantoms

No	Experiment	ResNet-Acc.	DenseNet-Acc.	ResNet-AUC	DenseNet-AUC
1	Train-Time Calibration with Calibration Phantom 1	93.69±0.70	94.36±0.97	0.983±0.003	0.984±0.004
2	Train-Time Calibration with Calibration Phantom 2	93.36±0.60	93.85±0.98	0.981 ±0.003	0.982±0.004
3	Test-Time Calibration with Calibration Phantom 1	98.73±0.30	98.96±0.32	0.999±4e-4	0.999±4e-4
4	Test-Time Calibration with Calibration Phantom 2	97.36±0.43	97.62±0.38	0.997±0.001	0.997±0.001

**TABLE VII:** *p*-Values for Table VI Based on Classification Accuracy

*No*	1	2	3	4
1	*	<.01	<.01	<.01
2	<.01	*	<.01	<.01
3	<.01	<.01	*	<.01
4	<.01	<.01	<.01	*

**TABLE VIII:** *p*-Values for Table VI Based on AUC Scores

*No*	1	2	3	4
1	*	<.01	<.01	<.01
2	<.01	*	<.01	<.01
3	<.01	<.01	*	<.01
4	<.01	<.01	<.01	*

**TABLE IX:** Stable Versus Hand-Free Calibration

No	Experiment	ResNet-Acc.	DenseNet-Acc.	ResNet-AUC	DenseNet-AJJC
1	Train-Time Calibration with Stable Calibration	93.69±0.70	94.36±0.97	0.983±0.003	0.984±0.004
2	Train-Time Calibration with Hand-Free Calibration	93.57±0.70	94.27±0.99	0.982±0.004	0.984±0.004
3	Test-Time Calibration with Stable Calibration	98.73±0.30	98.96±0.32	0.999±4e-4	0.999±4e-4
4	Test-Time Calibration with Hand-Free Calibration	98.66±0.33	98.90±0.35	0.999±6e-4	0.999±5e-4

**TABLE X:** *p*-Values for Table IX Based on Classification Accuracy

*No*	1	2	3	4
1	*	<.01	<.01	<.01
2	<.01	*	<.01	<.01
3	<.01	<.01	*	<.01
4	<.01	<.01	<.01	*

**TABLE XI:** *p*-Values for Table IX Based on AUC Scores

*No*	1	2	3	4
1	*	<.01	<.01	<.01
2	<.01	*	<.01	<.01
3	<.01	<.01	*	<.01
4	<.01	<.01	.07	*

**TABLE XII:** Different Transducers

No	Experiment	ResNet-Acc.	DenseNet-Acc.	ResNet-AUC	DenseNet-AUC
1	Train-Time Calibration with LI 1–5 vs L9–4	93.49±3.01	95.33±1.70	0.977±0.019	0.988±0.007
2	Test-Time Calibration with LI 1–5 vs L9–4	97.51 ±0.76	97.57±0.72	0.996±0.002	0.997±0.002
3	Train-Time Calibration with L9–4 vs L9–4	93.57±0.70	94.27±0.99	0.982±0.004	0.984±0.004
4	Test-Time Calibration with L9–4 vs L9–4	98.66±0.33	98.90±0.35	0.999±6e-4	0.999±5e-4

**TABLE XIII:** *p*-Values FOR Table XII Based on Classification Accuracy

*No*	1	2	3	4
1	*	<.01	.49	<.01
2	<.01	*	<.01	<.01
3	.13	<.01	*	<.01
4	<.01	<.01	<.01	*

**TABLE XIV:** *p*-Values for Table XII Based on AUC Scores

*No*	1	2	3	4
1	*	<.01	.84	<.01
2	<.01	*	<.01	<.01
3	.13	<.01	*	<.01
4	<.01	<.01	<.01	*

## Highlights

- The proposed method utilizes a transfer function to calibrate machine-related data mismatches for deep learning (DL)-based quantitative ultrasound.
- The results indicate that the proposed method can calibrate data between different ultrasound machines with high accuracy for DL purposes in ultrasound imaging.
- The proposed method can offer significant cost reduction in DL model development and enhances the understanding of the security aspects of DL models in ultrasound imaging.
